# Aryl hydrocarbon receptor pathway: Role, regulation and intervention in atherosclerosis therapy

**DOI:** 10.3892/mmr.2019.10748

**Published:** 2019-10-16

**Authors:** Kaixi Zhu, Qingqi Meng, Zhi Zhang, Tao Yi, Yuan He, Jing Zheng, Wei Lei

**Affiliations:** 1 Cardiovascular Medicine Center, Affiliated Hospital of Guangdong Medical University, Zhanjiang, Guangdong 524001, P.R. China; 2 Laboratory of Cardiovascular Diseases, Affiliated Hospital of Guangdong Medical University, Zhanjiang, Guangdong 524001, P.R. China; 3 Department of Orthopedics, Guangzhou Red Cross Hospital, Guangzhou, Guangdong 510000, P.R. China; 4 Department of Vascular, Thyroid and Breast Surgery, Affiliated Hospital of Guangdong Medical College, Zhanjiang, Guangdong 524001, P.R. China; 5 Department of Obstetrics and Gynecology, University of Wisconsin, Madison, WI 53715, USA

**Keywords:** AhR, atherosclerosis, inflammation, lipid deposition, endothelial cells

## Abstract

The aryl hydrocarbon receptor (AhR) is a ligand-activated transcription factor originally isolated and characterized as the dioxin or xenobiotic receptor. With the discovery of endogenous ligands and studies of AhR knockout mice, AhR has been found to serve an important role in several biological processes, including immune responses and developmental and pathological regulation. In particular, it has been considered as a new major player in cardiovascular diseases. Recent studies have revealed that the development of atherosclerosis is closely associated with AhR function. However, the roles of the AhR in the pathological development of atherosclerosis and atherosclerosis-associated diseases remain unclear. The current review presents the molecular mechanisms involved in the regulation of AhR expression during inflammation, oxidative stress and lipid deposition. Additionally, the role of the AhR in atherosclerosis and atherosclerosis-associated diseases is reviewed.

## 1. Introduction

**Figure 1.**
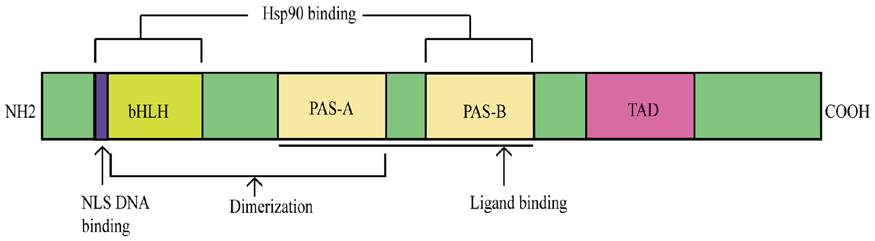
Structure of the AhR. The AhR consists of a bHLH/PAS domain and a TAD. AhR, aryl hydrocarbon receptor; bHLH, basic helix-loop-helix; PAS, PER-ARNT-SIM; TAD, transactivation domain; Hsp90, heat shock protein 90; NLS, nuclear localization sequence.

The aryl hydrocarbon receptor (AhR) is ligand-dependent and mediates nuclear receptors that react with heterologous substances of phases I and II (Fig. 1). The theory of polycyclic aromatic compound (PAC) metabolic reactions was postulated in the late 1950s (1). The carcinogenic dye 3-methylcholanthrene induces the synthesis of a specific enzyme that detoxifies 3-methylcholanthrene by promoting the synthesis of the liver microsomal enzyme P450, which is an aryl hydrocarbon hydroxylase (AHH) (1). Other carcinogens, including insecticides and phenobarbital, have a similar effect. This increase in synthesis meets the criteria of an adaptive response, as the upregulated enzyme oxidizes the PAH inducer when it is re-exposed within a short timeframe (2–4). Genetic studies have revealed that AHH is regulated by multiple alleles. These alleles were originally called Ah and used to describe the reaction of aromatic hydrocarbons (5).

**Figure 2.**
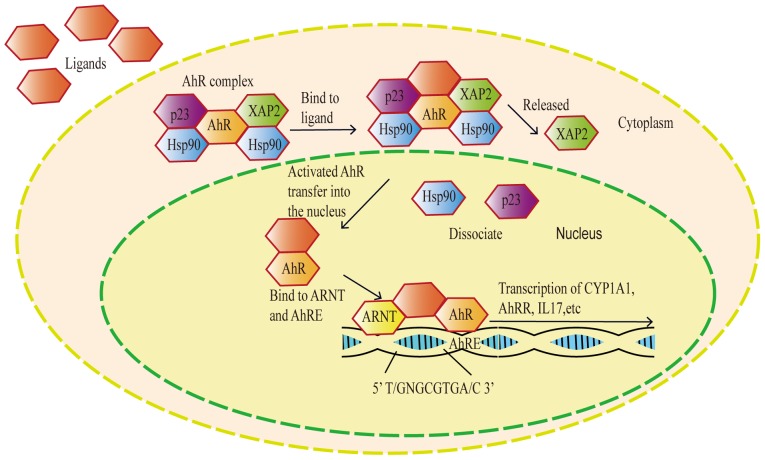
Model of the AhR signaling pathway. Upon binding to a ligand, the AhR is activated and enters the nucleus, where it binds to ARNT on AhRE and promotes transcription of downstream genes including CYP1A1 and IL-1. AhR, aryl hydrocarbon receptor; ARNT, aryl hydrocarbon receptor nuclear translocator; AhRE, aryl hydrocarbon response element; CYP1A1, cytochrome P450 family 1 subfamily A member 1; IL-1, interleukin 1; XAP2, aryl hydrocarbon receptor interacting protein; AHRR, aryl-hydrocarbon receptor repressor; IL-11, interleukin 17; Hsp90, heat shock protein 90; p23, prostaglandin E synthase 3.

The AhR belongs to the basic helix-loop-helix (bHLH) family and has a Per-Arnt-Sim (PAS) domain that binds to a variety of endogenous and exogenous chemicals. It binds specific auxiliary proteins, including heat shock protein 90 and hepatitis B virus X-associated protein, in the cytoplasm of resting state cells (6,7). When the AhR is transferred to the nucleus, it combines with the aryl hydrocarbon receptor nuclear translocator and triggers the transcription of several downstream genes, including cytochrome P450 family 1 subfamily A member 1 (CYP1A1) and cytochrome P450 family 1 subfamily B member 1 (CYP1B1), resulting in a variety of physiological and toxicological effects (8) (Fig. 2). Aryl hydrocarbon receptors are polymorphic. Known alleles include AhRb-1–3 and AhRd (9). The receptors have different affinities, however; all four proteins are alkaline and contain a bHLH as well as PAS and transactivation domains (10).

The sensitivity of AhR to 2,3,7,8-tetrachlorodibenzo-p-dioxin (TCDD) is species-specific, and its persistence in different organs *in vivo* varies according to the expression pattern of AhR in the specific organ (11). The AhR is most highly expressed in the human placenta, followed by the lungs, heart, pancreas and liver. Its lowest expression levels are in the kidney, brain and skeletal muscle (9). The AhR is transcribed from highly conserved sequences and plays a regulatory role in system development and physiological processes of different organs. Therefore, its expression is of great importance. The AhR is activated by the high-affinity exogenous ligands HAH and PAH as well as low-affinity endogenous ligands such as arachidonic acid, pyrene, and tryptophan and flavonoid derivatives (12–14). The endogenous ligands activate the AhR to participate in the regulation of cardiac functions, vascular development and blood pressure (15–19). In addition, the AhR signaling pathway senses changes in the circadian rhythm, oxygen tension and redox potential to regulate neural development and vacularization (20). For example, exposure to polycyclic aromatic hydrocarbons in cigarette smoke results in oxidative stress and the production of oxidized low-density lipoprotein (ox-LDL). ox-LDL accumulation in macrophages and smooth muscle-derived pro-inflammatory foam cells is a hallmark of atherosclerosis (21).

Studies using AhR^−/−^ mice have revealed that the AhR is a vital regulator of growth, development and material metabolism (22,23). Recent reports revealed that the AhR may exert harmful effects relating to endothelial dysfunction and immune disorders (24,25). AhR ligands activate the inflammatory axis in vascular endothelial cells to promote cell apoptosis and the inflammatory response (26).

The aim of the present review was to discuss the nature of the AhR, mediation of exogenous drugs, and potential targets for modification of cardiovascular genes. The role of the AhR receptor in the cardiovascular system, particularly the mechanism of action of AhR in atherosclerosis, is discussed in the present review. The role of the AhR in the development of novel therapeutic agents for the treatment of cardiovascular diseases is also presented.

## 2. AhR regulation in atherosclerosis pathogenesis

Atherosclerosis is a chronic inflammatory disease (27,28). The pathology of atherosclerosis can be summarized as follows. Foreign or endogenous substances, such as PAHs, PCBs and indoleamine 2,3-dioxygenase, cause oxidative stress, inflammation and the release of interleukin (IL) 1 and tumor necrosis factor (TNF) which stimulate chemokines, vascular endothelial cells, vascular endothelial cells, or neutrophils in the vascular interstitium (29,30). Inflammatory factors induce the oxidation of low-density lipoproteins which are phagocytized by macrophages which subsequently become lipid lines (31). The inflammatory factors induce the chemotaxis of monocytes in blood vessels into the stromal cells, where they differentiate into macrophages (32,33). This is the early development of atherosclerosis (34). Lipids (mainly cholesterol) are deposited in the intima of large and medium blood vessels (35,36). Smooth muscle cells and collagen fibers increase in number, and, secondary to necrosis, atheromatous plaques form. The plaques often cause different degrees of stenosis of the vascular lumen (37). Diseases with hardening of the blood vessel wall may present with ischemic changes in the end organs.

### 

#### AhR signaling pathway in inflammation and atherosclerosis

Atherosclerosis is an inflammatory immune disease; it's inflammatory etiology was first proposed by Ross (28). Nuclear factor κ-B (NF-κB) is a key signal transduction factor and plays a central role in inflammatory cytokine-mediated inflammatory responses. When cells are stimulated by various internal factors including SRC-1 and p300, the NF-κB signaling pathway is activated, and nuclear factors combine with the corresponding genes, thereby regulating the expression of target genes that magnify the inflammatory response, such as chemokines, inflammatory cytokines (including TNF-α, IL-1 and IL-6) and adhesion molecules [including intercellular cell adhesion molecule-1 and vascular cell adhesion molecule-1 (VCAM-1)] (29,38). In the cytoplasm, AhR competitively binds to the RELA proto-oncogene, NF-κB subunit (RELA) in NF-κB in a ‘tethered’ manner, preventing the AhR from combining with a required synergistic activator (39). By binding to the promoter of AhR-NF-κB1, RELA regulates the promoter sequence, affecting the expression of the AhR (40). In the coronary endothelium, whether the AhR signaling pathway exerts adverse effects on physiological functions through the NF-κB signaling pathway remains unreported (41).

**Figure 3.**
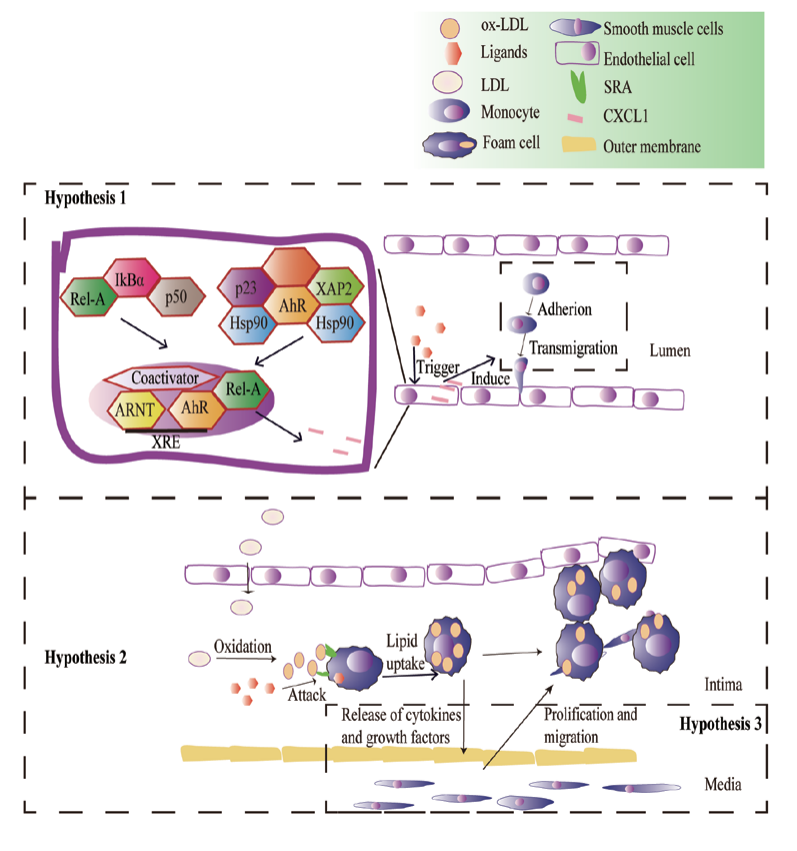
Role of AhR in the pathology of atherosclerosis. Hypothesis 1: Signaling of downstream inflammatory factors such as vascular cell adhesion molecule 1 via the AhR/nuclear factor-κB signaling pathway leads to monocyte chemotaxis. Hypothesis 2: AhR promotes macrophage absorption of ox-LDL and the formation of foam cells by mediating endogenous and exogenous ligands such as ox-LDL, lipopolysaccharide and 2,3,7,8-tetrachlorodibenzo-p-dioxin. Hypothesis 3: Increased proliferation of vascular smooth muscle cells is implicated in the occurrence of vascular complications. AhR, aryl hydrocarbon receptor; attack, pathological response induced by ligands; ox-LDL, oxidized low-density lipoprotein; p50, Rho guanine nucleotide exchange factor 7; RELA, RELA proto-oncogene NF-κB subunit; Ikbα, NF-κB inhibitor α; XAP2, aryl hydrocarbon receptor interacting protein; ARNT, aryl hydrocarbon receptor nuclear translocator; Hsp90, heat shock protein 90; p23, prostaglandin E synthase 3; CXCL1, C-X-C motif chemokine ligand 1; XRE, xenobiotic response element; SRA, scavenger receptor A.

There are three hypotheses underlying the AhR signaling pathways that mediate inflammation and promote atherosclerosis. The first hypothesis involves the signaling of downstream inflammatory factors such as VCAM-1 via the AhR/NF-κB signaling pathway, which leads to monocyte chemotaxis (42). Macrophages and monocytes are targeted by polycyclic aromatic hydrocarbons involved in the physiological and pathological processes of atherosclerosis (43). The second hypothesis postulates that the AhR promotes macrophages absorption of ox-LDL to form foam cells by mediating endogenous and exogenous ligands such as ox-LDL, lipopolysaccharides and TCDD. *In vitro* studies have revealed that cholesterol accumulation in foam cells caused by particulate matter-induced inflammation is an early sign of cardiovascular disease (30,44). However, the inhibitory effect of AhR inhibitors on foam cells and inflammation have not been investigated. It is believed that these mechanisms will be elucidated by extensive research of the AhR. The third hypothesis involves the increased proliferation of vascular smooth muscle cells (VSMCs), which is a critical factor in the occurrence of vascular complications. Yisireyili *et al* (45) exposed VSMCs to indoxyl sulfate, an agonist of AhR. Indoxyl sulfate induces VSMC proliferation via the activation of the AhR, the NF-κB signaling pathway and reactive oxygen species (ROS) production (Fig. 3).

#### AhR signaling pathway in oxidative stress and atherosclerosis

AhR mediates exogenous chemicals, such as TCDD and dioxin-like planar polychlorinated biphenyls (PCBs), and endogenous substances, including indoxyl sulfate and arachidonic acid, by activating NADPH oxidase to produce ROS that directly damages vascular endothelial cells. This may result in a cellular oxidative stress/antioxidant imbalance that leads to cell damage and reduces the integrity of the vascular endothelium (46,47). Previous studies have revealed that ROS mediate the transcription of specific genes, such as NF-κB, which mediate the transcription of inflammatory inducible nitric oxide synthase (44,48).

#### AhR signaling pathway in lipid deposition and atherosclerosis

Lipid deposition is an essential external condition for foam cell formation (49). The current clinical treatment mainly relies on lipid-lowering drugs such as atorvastatin and fenofibrate (50). Lipid metabolism mainly occurs in the liver. It has been validated by recent studies that the AhR not only detoxifies, but also regulates lipid metabolism in the liver (51). Environmental pollutants such as TCDD and benzo(a)pyrene (BP) inhibit the expression of NPC intracellular cholesterol transporter 1 in an AhR-dependent manner, promoting lipid deposition (52).

Lipid deposition can be induced by several AhR ligands. TCDD is a classical AhR ligand. A previous study revealed that α-endosulfan and 2,3,7,8-TCDD jointly downregulate the expression of glucose- and lipid-associated genes in the liver, such as nuclear receptor subfamily 1 group H member 4 and nuclear receptor binding factor 2 (53). Lipoxin A4 (LXA4), an endogenous ligand of AhR, is induced by homocysteine in patients with hyperhomocysteinemia. LXA4 promotes the binding of the AhR to the promoter of CD36 in hepatocytes and promotes CD36 expression, which increases the uptake of fatty acids and lipid accumulation by hepatocytes (54). Previous studies revealed that AhR activation affects the systemic metabolic functions of mice, including suppressed tricarboxylic acid cycle, disrupted lipid metabolism, amino acids metabolism, glycogenolysis, gluconeogenesis, thereby increased hepatic lipogenesis, and promotedinflammatory signaling pathways (23,55,56).

AhR knockout mice are widely used to study the role of the AhR in physiological functions. Activation of AhR protects against fatty liver induced by insulin resistance by activating fibroblast growth factor 21 (FGF21) to regulate lipid and energy metabolism in such mice (57). AhR knockout mice have increased levels of energy metabolism compared with normal mice, which protects against insulin resistance, hepatic steatosis, obesity and inflammation caused by a high-fat diet (HFD) (23). By contrast, the AhR protects against hepatic steatosis induced by a HFD and subsequent lipotoxicity. The AhR protects against fatty liver induced by insulin resistance by activating FGF21. The endocrine signaling pathway of AhR and FGF21 suggests that AhR is a crucial environmental modifier that combines signals from chemical exposure to regulate lipid and energy metabolism (57). *In vivo* experiments have revealed that locked nucleic acid 29, an inhibitor of microRNA (miR)-29, inhibits lipid deposition in the liver, and whole-genome analysis demonstrated increased AhR and sirtuin1 expression (58). AhR is a direct target gene of miR-29. Therefore, it may be an alternative therapeutic target for treating metabolic disorders such as dyslipidemia (58). PCB 153, mediated by AhR, can be considered as a ‘secondary strike’ mechanism for obesity/non-alcoholic fatty liver disease in the context of a HFD (59).

## 3. Clinical research about AhR and atherosclerosis-associated diseases

Studies investigating the association of the AhR signaling pathway and its downstream genes, glutathione S-transferase μ1 (GSTM1) and glutathione S-transferase θ1 (GSTT1), with the risk and complications of atherosclerosis-associated diseases have yielded inconclusive results (60,61). Recent studies revealed that AhR may be associated with atherosclerosis-associated diseases, including coronary artery disease (CAD), ischemic stroke and type 2 diabetes mellitus (T2DM) (62–64) (Table I). The current review presents clinical research to reveal their association.

### 

#### Role of the AhR in the occurrence and development of CAD

CAD may result in mortality and is associated with atherosclerosis and thrombosis (65). There is no clinically relevant research on AhR gene polymorphisms and atherosclerosis, to the best of our knowledge, and few studies on AhR gene polymorphism and CAD (66–68). Receiver operating characteristic analysis of 939 patients with confirmed CAD and 868 normal subjects indicated that the AhR is a potential marker for objective measurement and evaluation of CAD in addition to other cardiac markers, such as creatine kinase-MB (69). Genotype frequencies of AhR rs2066853 reveal significant differences between CAD and control subjects, and hyperlipidemia and smoking significantly increased the risk of CAD associated with AhR polymorphism (69). Furthermore, the four subtypes of CAD with varying severity show significant differences in the distribution of AhR variants (70). Previous studies have investigated the association of CAD and the downstream mediators of the AhR signaling pathway, CYP1A1, GSTT1 and GSTM1, that mediate the metabolism of allogenic toxic substances (Table I). Previous studies in China have demonstrated significant associations of CYP1A1, GSTM1, GSTT1 and peroxisome proliferator activated receptor γ with CAD, particularly in smokers (70–72).

A cross-sectional study in Croatia included 252 adult subjects with suspected exposure to PAHs; it was revealed that CYP1A1, GSTM1 and GSTT1 gene polymorphisms had no association with the risk of CAD (73).

Previous studies in India have demonstrated an association between GSTM1/GSTT1/glutathione S-transferase π1 (GSTP1) polymorphism, coronary heart disease and blood lipid parameters (74–76). These findings suggest that blood lipid parameters in patients with coronary angiography are significantly associated with GSTM1/T1/P1 genotype distribution and GSTT1 deletion polymorphisms. However, a case-control study in the Republic of Korea revealed that GSTM1/T1 had no effect on the degree of lumen stenosis in CAD (77). Smokers carrying a GSTM1/T1 mutation have a higher risk of CAD (77).

Regarding the association between genetic polymorphisms of GSTM1 and smoking-related CAD, smokers with the GSTM1 null genotype have a greater risk of CAD compared with non-smokers with the GSTM1-positive genotype [odds ratio (OR), 2.07; 95% confidence interval (CI), 1.06–4.07]. The association between genetic polymorphisms of GSTT1 and smoking related-CAD smoking shares the same tendency as that for GSTM1 (OR, 2.00; 95% CI, 1.05–3.84). The association between GSTM1 and GSTT1 null genotypes in smoking-related CAD was also augmented when genetic polymorphisms of GSTM1 and GSTT1 were considered simultaneously (OR, 2.76; 95% CI, 1.17–6.52) (77). On the basis of several epidemiological studies, AhR downstream genes are significantly associated with CAD, particularly in smokers with the GSTT1/M1 knockout gene (78–80).

AhR mRNA and the mRNA level of its allele are higher in the peripheral blood of patients with CAD compared with controls (69). Smoking increases the risk of CAD in patients with AhR gene polymorphisms. This may be due to the aromatic hydrocarbons present in smoke which cause lipid metabolism through the AhR signaling pathway (81). Furthermore, nicotine exposure induces VCAM-1, matrix metalloprotein (MMP)-2 and MMP-9 production in VSMCs and macrophages (82) and promotes vascular oxidative stress, leading to vascular damage (83).

#### AhR expression and polymorphisms are associated with the risk of ischemic stroke

Ischemic stroke is regarded as a potentially fatal disease (84) that occurs when there is a sudden decrease of blood supply to brain tissue, resulting in ischemia and hypoxia. Although cerebral and myocardial infarctions have different sites, the pathological mechanism is atherosclerosis (85). Therefore, cerebral infarction may be associated with vascular inflammation and oxidative stress; AhR downstream genes, such as CYP1A1, GSTT1 and GSTM1, may serve important roles in the pathogenesis (86) (Table I).

A case-control study in Turkey, which included 226 patients with ischemic stroke and 113 controls, showed significant differences between 6235C allele and the risk of ischemic stroke, particularly in smokers and patients with hypertension (87).

Moon *et al* (86) investigated 353 patients with cerebral infarction and 376 controls. A significantly larger number of patients with cerebral infarction had the CYP1A1 gene 3′-flanking region (T6235C) compared with the controls (P=0.017; OR, 1.44; 95% CI, 1.07–1.94). Analysis of gene-gene interactions showed that the GSTM1 null genotype increased the cerebral infarction risk in carriers of the CYP1A1 C allele (P=0.015; OR, 1.47; 95% CI, 1.08–2.00) (86).

To investigate the association between stroke and polymorphism of the CYP1A1 gene, Sultana *et al* (88) selected 215 patients with ischemic stroke and 162 age-matched controls. The results indicated that ischemic stroke had a significant association with the CYP1A1 genotype ‘CC’ (P=0.01; OR, 5.14; 95% CI, 1.14–23.14) in south Indian population, whereas Zhang *et al* (89) showed that CYP1A1 decreased the risk of disease in the eastern Han of China, and this contradiction showed CYP1A1 gene to display distinct alleles distribution among populations. In conclusion, CYP1A1 was shown to be significantly associated with ischemic stroke in certain clinical studies; however, further investigation is required to verify this association.

#### Association of AhR and its downstream genes with T2DM

T2DM, a metabolic disease, is associated with oxidative stress and chronic inflammation in adipose tissue (90). Previous epidemiological studies have revealed associations between oxidative stress-associated enzymes, such as GSTT1 and GSTM1, and diabetes (Table I). GSTT1 and GSTM1 are AhR downstream genes.

A case-control study in India revealed that GSTM1 and GSTT1 are associated with gene polymorphism-associated fat mass and obesity. GSTM1-positive and GSTM1 null genotypes had significant associations with T2DM, but there was no significant association with FTO α-ketoglutarate-dependent dioxygenase polymorphism (91). Other epidemiological studies revealed that GSTM1 and GSTT1 did not have significant effects on T2DM (92,93). Hori *et al* (94) did not reveal a significant association between T2DM and GSTT1/M1 gene alleles, but the incidence rate of T2DM in GSTT1 and GSTM1 null genotypes was 1.5-fold higher than that in GSTM1 and GSTT1 positive genotypes. The aforementioned clinical studies demonstrated that GSTT1/M1 and diabetes are not highly associated and that further investigation is required to determine their associations.

## 4. Conclusion

**Figure 4.**
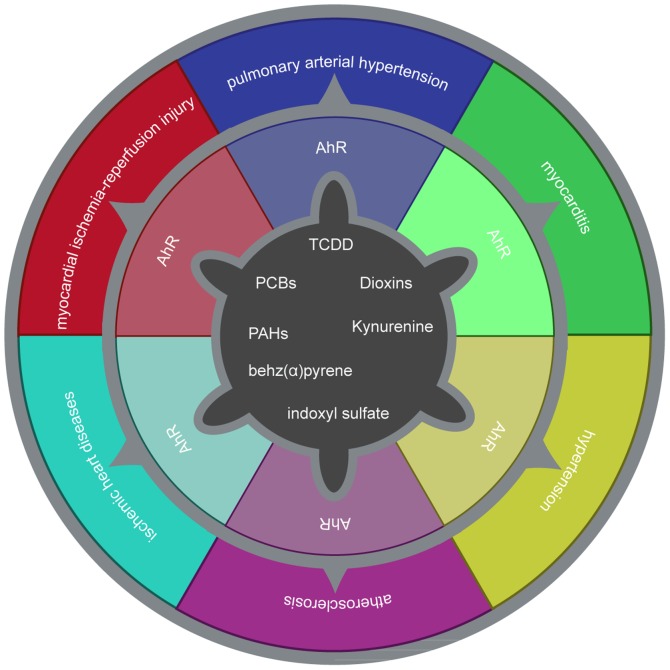
Relationship between AhR and cardiovascular disease.

The current review presented the association between the AhR and inflammation, oxidative stress, lipid infiltration and atherosclerosis. The AhR is closely associated with cardiovascular disease in terms of cardiac function, vascular development and blood pressure regulation (Fig. 4). In certain atherosclerosis-associated diseases, the AhR may serve a role as an oxidative stress signal transmitter. The AhR may be a potential target for the clinical treatment of cardiovascular disease. However, some important questions remain unanswered. The regulation of the AhR at the gene level has not been elucidated in humans. There are currently no drugs targeting the AhR in the clinic. The AhR is associated with other signaling pathways, including the Wnt and E2 factor signaling pathways, and further basic experiments are required to elucidate the roles of the AhR. The identification of novel endogenous ligands and the application of AhR knockout mice may clarify the role, regulation and intervention of the AhR in the treatment of atherosclerosis.

## Figures and Tables

**Table I.** Clinical studies related to the association of AhR, CYP1A1 and GST genetic polymorphisms with atherosclerosis-related diseases.

A, Studies related to AhR and CYP1A1 polymorphisms					

		AhR (NM_001621.5) CYP1A1 (KR710640.1)			
			
Author, year	Atherosclerosis-associated disease	Country	Polymorphism	Major observation	(Refs.)
Huang *et al*, 2015	CAD	China	AhR allele	Significant association of AhR rs2066853 with CAD, particularly among smokers and patients with hyperlipidemia.	(69)
Peng *et al*, 2017		China	CYP1A1 allele	Significant association of rs4886605, rs1048943 and rs4646903 with CAD, particularly among smokers.	(70)
Zhang *et al*, 2017		China	CYP1A1 allele	Polymorphism in PPARG allele and interaction with CYP1A1 allele were associated with increased CAD susceptibility.	(71)
Pašalić and Marinković, 2017		Croatia	CYP1A1 m1/m2/m4	No association between polymorphisms and markers of higher risk of CAD, such as lipid parameters in the general population.	(73)
Demirdöğen *et al*, 2013	Ischemic stroke	Turkey	CYP1A1 allele	The 6235C allele significantly increased the susceptibility of smokers to ischemic stroke compared with non-smokers.	(87)
Moon *et al*, 2007		Korea	CYP1A1 allele	C allele carriers in CYP1A1have a higher risk of cerebral infarction	(86)
Sultana *et al*, 2011		India	CYP1A1 allele	Different genotypes showed different risk of stroke. The order was CC homozygotes (P=0.01; OR, 5.14; 95% CI, 1.14–23.14), TT (P=0.25; OR, 0.78; 95% CI, 0.51–1.19), TC (P=0.85; OR, 1.04; 95% CI, 0.67–1.60).	(88)

**B, Studies related to GST polymorphisms**					

		**GST GSTT1(CR456499.1)/M1(CR541868.1)/P1(CR450361.1)**			
			
**Author, year**	**Atherosclerosis-associated disease**	**Country**	**Polymorphism**	**Major observation**	**(Refs.)**

Zhang and Zhang, 2014	CAD	China	GSTM1 deletion	Significant association of GSTM1 with CAD, particularly among smokers.	(72)
Pašalić and Marinković, 2017		Croatia	GSTM1/T1 deletions	No significant difference in GSTT1/GSTM1 gene polymorphism in patients with CAD.	(73)
Ramprasath *et al*, 2011		India	GSTM1/T1 deletion GSTP1 allele	All three variants may contribute to the development of T2DM and the GSTT1 variant was involved in the development of T2DM-associated CAD complications.	(76)
Mir *et al*, 2016		India	GSTM1/T1 deletions	Patients with the GSTM1 null genotype have increased risk of CAD compared with patients with the GSTT1 null genotype (OR, 2.28), particularly among smokers.	(74)
Bhat *et al*, 2016		India	GSTM1/T1 deletions	The combined analyses of the GST genotypes showed GSTM1 null genotype significant increased risk of CAD compared with the healthy controls.	(75)
Kim *et al*, 2008		Korea	GSTM1/T1 deletion	Significant association of GSTM1 and GSTT1 with smoking-associated CAD.	(77)
Moon *et al*, 2007	Ischemic stroke	Korea	GSTM1/T1 deletions	The GST1 null genotype increased the relative risk for cerebral infarction in the subjects with the CYP1A1 C allele than those without.	(86)
Raza *et al*, 2014	T2DM	India	GSTT1/M1 alleles	Patients with GSTM1 positive (P=0.046) and GSTM1 null (P=0.046) have significant associations with T2DM.	(91)
Etemad *et al*, 2016		Malaysia	GSTT1/M1 alleles	The groups showed no significant difference in terms of allele frequencies and GST polymorphism genotype (P=0.224 and 0.119).	(92)
Porojan *et al*, 2015		Romania	GSTM1/T1 gene alleles	GSTT1 and GSTM1 had no effect on T2DM independently; however, the combined GSTM1/GSTT1 null genotypes were statistically significantly higher in T2DM patients compared with control subjects.	(93)
Hori *et al*, 2007		Japan	GSTT1/M1 gene alleles	Although each null genotype was not significantly associated with T2DM, the incidence of T2DM in patients with the GSTT1 and GSTM1 null genotypes was 1.5-fold higher than the GSTT1 and GSTM1 positive genotypes.	(94)

A χ^2^ test and a 2×2 contingency table were applied to the calculation of P-values. P<0.05 were considered to indicate a statistically significant difference. CYP1A1, cytochrome P450 family 1 subfamily A member 1; GST, glutathione S-transferase; AhR, aryl hydrocarbon receptor; CAD, coronary artery disease; OR, odds ratio; CI, confidence interval; T2DM, type 2 diabetes mellitus; GSTM1, glutathione S-transferase µ1; GSTT1, glutathione S-transferase θ1; GSTP1, glutathione S-transferase π1.

## Data Availability

Not applicable.

## Abbreviations

AhR: aryl hydrocarbon receptor
PAC: polycyclic aromatic compound
AHH: aryl hydrocarbon hydroxylase
bHLH: basic helix-loop-helix
PAS: Per-Arnt-Sim
VSMCs: vascular smooth muscle cells
ROS: reactive oxygen species
FGF21: fibroblast growth factor 21
CAD: coronary artery disease
T2DM: type 2 diabetes mellitus
CYP1A1: cytochrome P450, family 1, subfamily A, polypeptide 1
IL: interleukin
ox-LDL: oxidized low-density lipoprotein
NF-κB: nuclear factor kappa-B
TNF: tumor necrosis factor
VCAM-1: vascular cell adhesion molecule-1
PCBs: planar polychlorinated biphenyls
TCDD: 2,3,7,8-tetrachlorodibenzo-p-dioxin
MMP: matrix metalloproteinase
LXA4: lipoxin A4
HFD: high-fat diet
OR: odds ratio
CI: confidence interval
